# Glaucomatous vertical vessel density asymmetry of the temporal raphe detected with optical coherence tomography angiography

**DOI:** 10.1038/s41598-020-63931-7

**Published:** 2020-04-22

**Authors:** Yuji Yoshikawa, Takuhei Shoji, Junji Kanno, Hisashi Ibuki, Robert N. Weinreb, Makoto Araie, Kei Shinoda

**Affiliations:** 1 Saitama Medical University, Department of Ophthalmology, Saitima, Japan; 2 Kanto Central Hospital of Mutual Aid Association of Public School Teachers, Tokyo, Japan; 3 Hamilton Glaucoma Center, Shiley Eye Institute, and Viterbi Family Department of Ophthalmology, University of California San Diego, La Jolla, CA USA

**Keywords:** Ocular ischemic syndrome, Optic nerve diseases, Eye manifestations

## Abstract

Changes in retinal vasculature and ocular circulation may play an important role in the glaucoma development and progression. We evaluated the vertical asymmetry across the temporal raphe of the deep retinal layer vessel density, using swept-source optical coherence tomography angiography (SS-OCTA), and its relationship with the central visual field (VF) loss. Thirty-four eyes of 27 patients with open-angle glaucoma were included. SS-OCTA macular scanning was performed within a 3 × 3 mm (300 × 300 pixels) volume, centred on the fovea. The relationships between the vertical asymmetrical deep retinal vessel density reduction (ADRVD) across the temporal raphe and various ocular parameters were analysed. Twenty-two glaucomatous eyes with ADRVDs had central VF loss. Contrarily, ADRVDs were not found in any of the 12 eyes without central VF loss. Thirteen eyes (59.1%) with central VF loss had ADRVDs topographically corresponding to the central VF loss and macular ganglion cell complex thinning. The glaucomatous eyes with ADRVDs exhibited inferior rather than superior central VF loss (*P* = 0.032). Thus, ADRVD specifically indicates the glaucomatous central visual loss. Further analysis of ADRVD may improve our understanding on glaucoma pathogenesis, offering new treatment insights.

## Introduction

Glaucoma is a progressive optic neuropathy constituting the world’s leading cause of irreversible blindness^1–4^. The disease is characterised by a progressive loss of retinal ganglion cells and their axons. Although mechanical stress related to intraocular pressure (IOP) is a principal risk factor for the disease, ocular blood flow may also have an important role in the development and progression of glaucoma^5–8^.

Several fundus fluorescein angiography studies have shown that glaucoma may be associated with changes in the retinal vasculature and ocular circulation^9–11^. Optical coherence tomography angiography (OCTA) is an *in vivo*, non-invasive technique for imaging retinal blood vessels. Information from OCTA studies may enhance our understanding of how ocular blood flow and the retinal microvasculature influence glaucoma development and progression. Additionally, OCTA retinal vasculature imaging does not require a dye injection^12–14^. Previous OCTA investigations have shown that glaucoma is associated with ocular microvasculature changes; changes in the optic nerve head vessel density (VD)^15–18^ superficial peripapillary VD^19,20^, macular VD^16,20–22^, and foveal avascular zone area^23,24^, and the parapapillary choroidal microvascular dropout^25,26^.

The OCTA technique is a promising tool for the microvasculature analysis, but OCTA images may contain critical image artefacts (i.e. projection artefacts [PAs]) that may compromise the deep retinal layer (DRL) image analysis^27^. However, the PA removal software has recently been developed, and several studies have successfully used it^28,29^. Using the DRL OCTA images that have had PAs removed, we have noticed that patients with glaucoma often exhibit vertical asymmetry of vessel-density loss across the temporal raphe.

To the best of our knowledge, no prior studies have evaluated the macular microvasculature structural changes in the DRL and the temporal raphe area. Therefore, we aimed to evaluate the OCTA findings and characterise the vertical asymmetrical loss of deep retinal vessel density in glaucomatous eyes.

## Results

**Figure 1:**
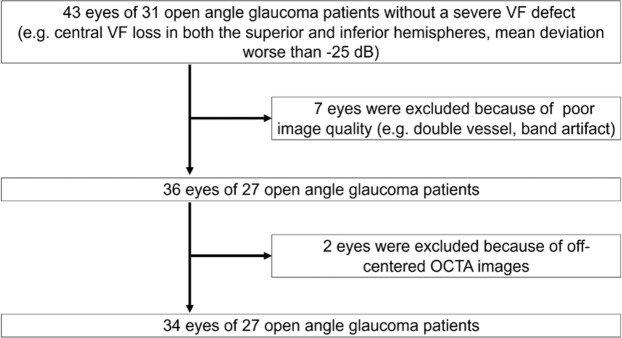
Flow chart representing study enrolment. VF, visual field; OCTA, optical coherence tomography angiography.

A total of 43 eyes from 31 patients with glaucoma were included for analysis. Two and seven eyes were excluded because of the off-centre and poor-quality images, respectively. There was no image with severe segmentation error. Therefore, 34 eyes from 27 patients (mean age, 63.7 ± 12.9 years; sex, 12 men and 19 women) were ultimately included in the analyses (Fig. 1).

Table 1 summarises the ocular and demographic characteristics of the included participants. The mean deviation (MD) and pattern standard deviation (PSD) values were −10.5 (6.4) dB and 10.1 (4.3) dB, respectively. The Cohen’s Kappa values for the asymmetrical deep retinal vessel density reduction (ADRVD) determination of intra-examiner and inter-examiner reliabilities were 0.879 (0.715–1.000) and 0.706 (0.472–0.940), respectively. There were 13 and 21 eyes with and without ADRVD. Table 1 shows the demographic and ocular characteristics of the included glaucomatous eyes. There were no statistically significant differences between the glaucomatous eyes with and without ADRVD. However, four of the 21 participants without ADRVD (19.1%) had diabetes, where no one with ADRVD had diabetes (*P* = 0.041).

**Table 1:** Demographic and ocular characteristics of the included glaucomatous eyes.

	Without ADRVD	With ADRVD	*P*
Participants, n	18	9	
Age, mean (SD), years	65.5 (13.5)	58.7 (12.8)	0.220*
Female, n (%)	10 (55.6)	4 (44.4)	0.586**
Eyes, n	21	13	
Age, mean ± SD, years	65.8 (13.0)	60.4 (12.5)	0.242*
Female, n (%)	13 (61.9)	6 (46.2)	0.369**
Ocular characteristics			
LogMAR BCVA, mean (SD)	0.00 (0.12)	0.01 (0.10)	0.662*
SE, mean (SD), dioptres	−1.7 (5.0)	−4.1 (2.7)	0.124*
IOP, mean (SD), mmHg	16.0 (9.9)	13.2 (3.8)	0.342*
Axial length, mean (SD), mm	25.1 (1.3)	25.7 (1.7)	0.232*
RNFL thickness, mean (SD), µm	61.5 (19.7)	61.5 (19.7)	0.689*
mGCL thickness, mean (SD), µm	19.4 (2.7)	19.0 (2.9)	0.668*
PSD, mean (SD), dB	9.1 (4.7)	11.7 (3.0)	0.086*
MD, mean (SD), dB	−9.3 (6.8)	−12.4 (5.6)	0.177*
Systemic characteristics			
History of hypertension, n (%)	7 (33.3)	7 (53.9)	0.238**
History of diabetes, n (%)	4 (19.1)	0 (0.0)	0.041**

ADRVD, vertical asymmetrical deep retinal vessel density reduction; VF, visual field; SD, standard deviation; logMAR, logarithm of the minimum angle of resolution; BCVA, best-corrected visual acuity; SE, spherical equivalent; IOP, intraocular pressure; PSD, pattern standard deviation; MD, mean deviation; RNFL, retinal nerve fibre layer; mGCL, macular ganglion cell layer.

*P value based on unpaired t-tests. **P value based on chi-square tests.

**Figure 2:**
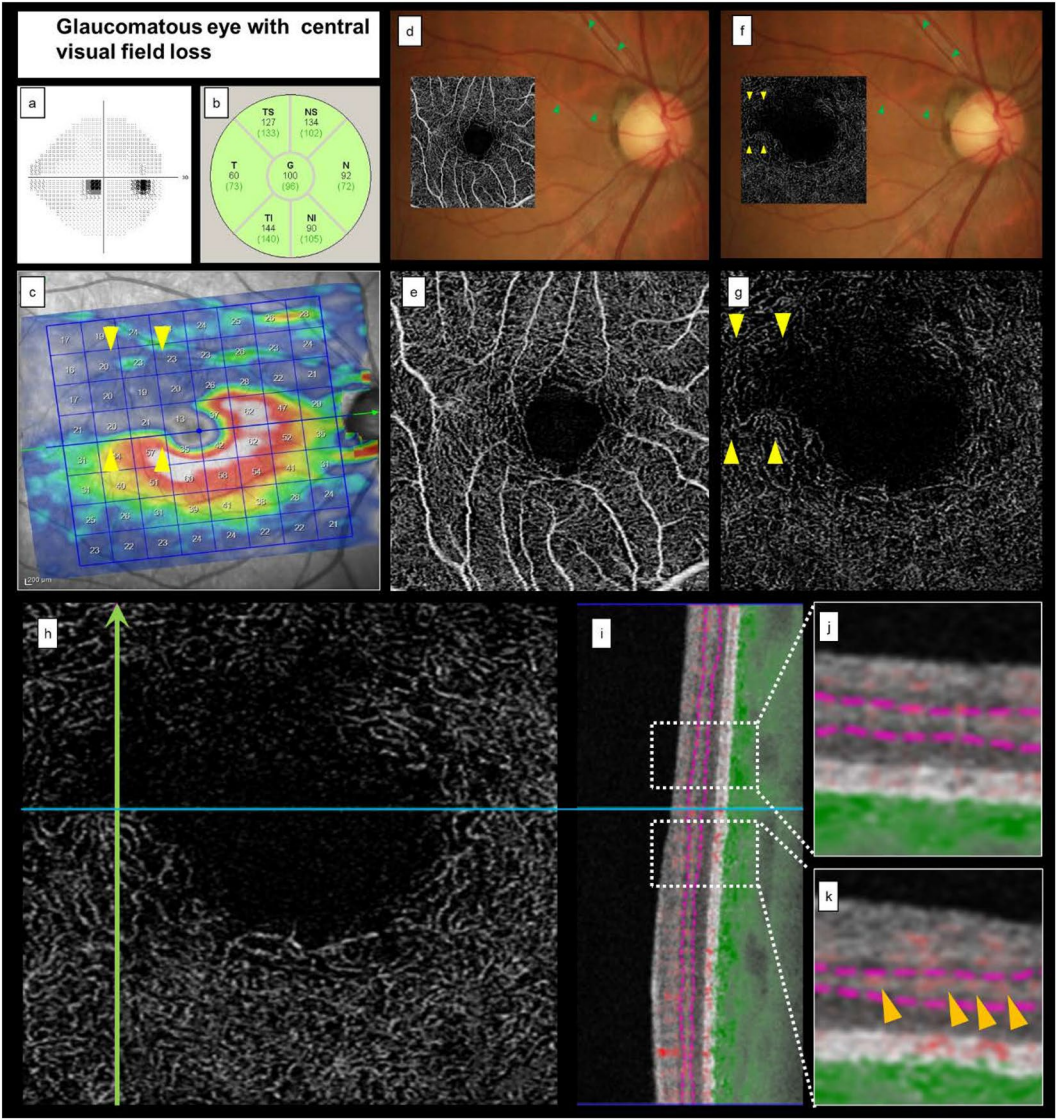
Representative case of a glaucomatous eye with central visual field loss. (**a–c)** cpRNFL thinning did not occur, but superior mGCL thinning was presented, indicating VF loss. (**d,f)** A fundus photograph superimposed on an OCTA *en face* image shows glaucomatous optic neuropathy and an RNFL defect. (**d,e)** The colour fundus photograph and the corresponding SRL OCTA projection do not indicate microvascular changes. (**f,g)** The DRL OCTA image shows ADRVD (yellow arrowheads) and locations of the corresponding ADRVD, mGCL thinning, and central VF loss. (**h,i)** An OCTA image shows the location, in which a vertical line scan (**h**, green arrow) of the microvasculature was obtained. (**j,k)** Magnified views are also shown of the white squares. The red shading indicates the blood flow. The SRL microvascular signal is intact, but the DRL microvascular signal is reduced (**lll** arrow heads). cpRNFL, circumpapillary retinal nerve fiver layer; mGCL, macular ganglion cell layer; VF, visual field; OCTA, optical coherence tomography angiography; ADRVD, vertical asymmetrical deep retinal vessel density reduction; DRL, deep retinal layer; SRL, superficial retinal layer.

Figure 2 shows the VF results and an image of a glaucomatous eye with inferior central VF loss, superior macular ganglion cell layer (mGCL) thinning, and ADRVD (visible on OCTA *en face* images, as a lower VD in the DRL than in the superficial retinal layer [SRL]).

The prevalence of ADRVD in the glaucomatous eyes with and without central VF loss was examined (Table 2). Thirteen and zero of the 22 examined eyes (59.1%) with central and without central VF loss had an ADRVD, respectively (*P* < 0.001). Similar results were obtained when the data from only one randomly chosen eye were used from each participant (all included eyes met the study inclusion/exclusion criteria, Table 2).

**Table 2:** Effect of the microvascular reduction vertical asymmetry across the temporal raphe on central visual field loss.

	Without ADRVD	With ADRVD	*P*
Eyes, n	21	13	
With central VF loss, n (%)	9 (42.9)	13 (100)	<0.001*
Without central VF loss, n (%)	12 (57.1)	0 (0.0)	
One eye from each participant, n	18	9	
With central VF loss, n (%)	7 (38.9)	9 (100)	<0.001/8
Without central VF loss, n (%)	11 (61.1)	0 (0.0)	

ADRVD, vertical asymmetrical deep retinal vessel density reduction; VF, visual field.

*P* values are based on chi-square tests.

(*) Statistically significant.

Additionally, there was a significantly higher ADRVD frequency in the glaucomatous eyes with inferior than in those with superior central VF loss (87.5% vs. 42.9%, *P* = 0.032; Table 3). In all cases, ADRVD area topographically corresponded to the mGCL thinning area in the macular and temporal raphe regions. No other significant differences were identified between the glaucomatous eyes or the patients with superior and inferior VF loss (Table 3). In eyes with central VF loss, the SRL parafoveal VD was not significantly different between the affected and unaffected hemispheres, even when an ADRVD was presented (non-affected, 37.3 ± 5.5; affected, 35.2 ± 6.3 *P* = 0.092). However, the DRL parafoveal VD was significantly lower in the affected than in the unaffected hemisphere when an ADRVD was presented (non-affected, 26.3 ± 8.2; affected, 20.6 ± 6.2 *P* = 0.001).

**Table 3:** Demographic and ocular characteristics of patients with glaucoma with superior and inferior central visual field loss.

	Superior VF loss	Inferior VF loss	*P*
Eyes, n	14	8	
Age, mean (SD), years	67.1 (13.6)	58.4 (11.4)	0.140*
Female, n (%)	8 (57.1)	4 (50.0)	> 0.999**
Ocular characteristics			
LogMAR BCVA, mean (SD)	0.01 (0.13)	0.01 (0.10)	0.936*
SE, mean (SD), dioptres	-2.2 (2.7)	−1.9 (7.9)	0.887*
IOP, mean (SD), mmHg	13.2 (3.4)	13.2 (4.6)	0.981*
Axial length, mean (SD), mm	25.0 (1.6)	26.1 (1.5)	0.146*
RNFL thickness, mean (SD), µm	54.9 (14.0)	60.3 (24.4)	0.514*
mGCL thickness, mean (SD), µm	18.2 (2.7)	19.9 (3.0)	0.202*
PSD, mean (SD), dB	12.4 (2.7)	11.0 (3.3)	0.298*
MD, mean (SD), dB	−14.0 (4.0)	−12.2 (6.4)	0.425*
ADRVD, n (%)	6 (42.9)	7 (87.5)	0.032**
Systemic characteristics			
History of hypertension, n (%)	7 (50.0)	5 (62.5)	0.323**
History of diabetes, n (%)	2 (14.3)	1 (12.5)	0.907**

ADRVD, vertical asymmetrical deep retinal vessel density reduction; VF, visual field; SD, standard deviation; logMAR, logarithm of the minimum angle of resolution; BCVA, best-corrected visual acuity; SE, spherical equivalent; IOP, intraocular pressure; PSD, pattern standard deviation; MD, mean deviation; RNFL, retinal nerve fibre layer; mGCL, macular ganglion cell layer.

*P value based on unpaired t-tests.

**P value based on chi-square tests.

## Discussion

The presence of ADRVD and central visual field defect may be clinically relevant, as central visual dysfunction may hinder daily activities, such as mobility, driving, and reading. Moreover, the development of visual field loss that verges on fixation (i.e. threatened visual field loss) is of concern when managing patients with glaucoma.

**Figure 3:**
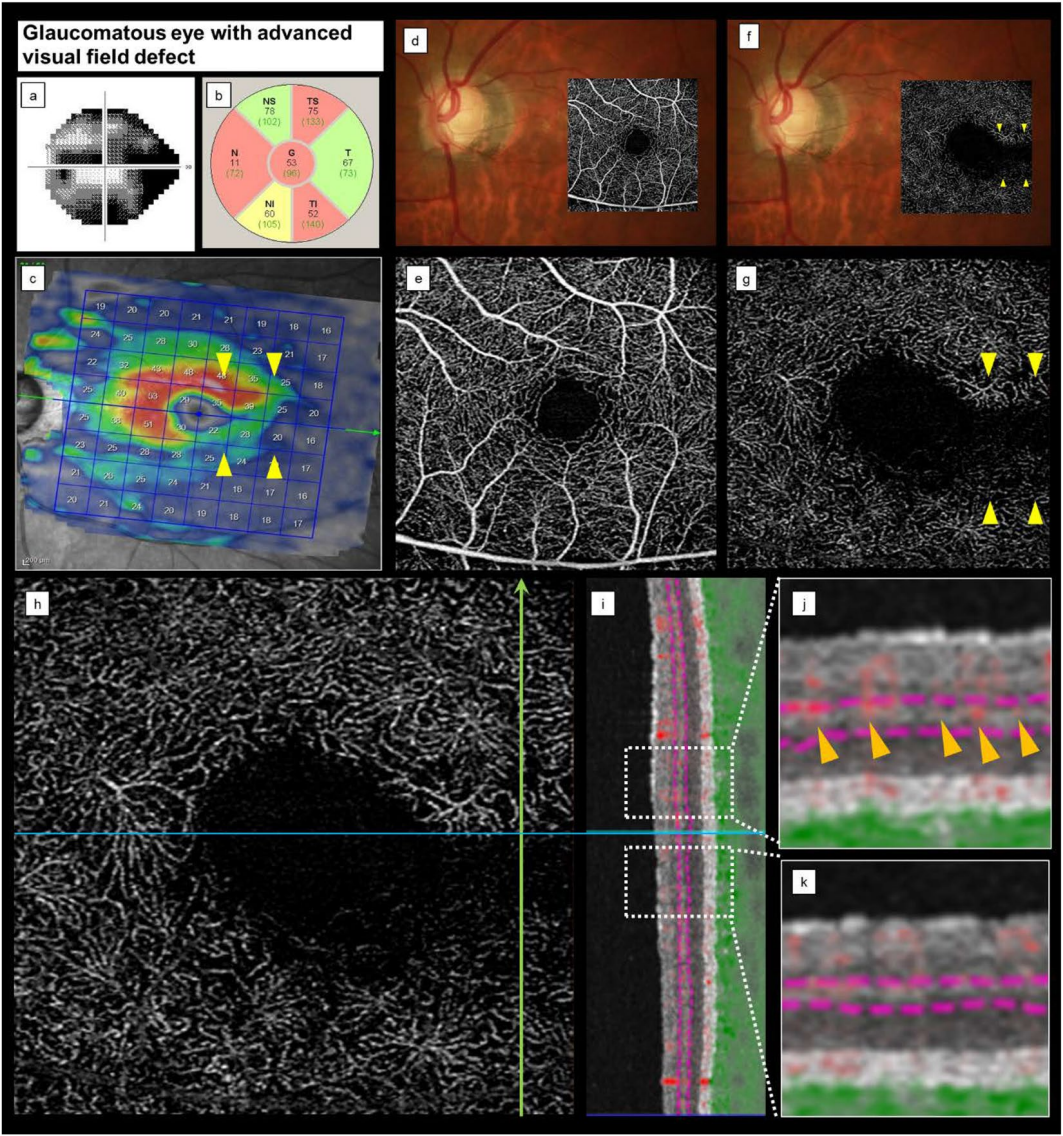
Representative case of a glaucomatous eye with advanced visual field loss. (**a**) VF testing shows an advanced VF loss. (**b,c**) A fundus photograph superimposed on an OCTA *en face* image shows superior and inferior temporal cpRNFL and inferior mGCL thinning corresponding to VF loss. These findings indicate glaucomatous optic neuropathy. (**d,e**) The colour fundus photograph and the SRL OCTA image do not show signs of microvascular changes. (**f,g**) The DRL OCTA image shows apparent vertical ADRVD (yellow arrowheads) and the corresponding ADRVD, GCL thinning, and central VF loss locations. (**h,i**) An OCTA image shows the location, in which a vertical line scan (h, green arrow) of the microvasculature was obtained. (**j,k**) Magnified views are also shown by the white squares. The red shading indicates the blood flow. The SRL microvascular signal is intact, but the DRL microvascular signal is reduced (yellow arrowheads). cpRNFL, circumpapillary retinal nerve fiver layer; mGCL, macular ganglion cell layer; VF, visual field; OCTA, optical coherence tomography angiography; ADRVD, vertical asymmetrical deep retinal vessel density reduction; DRL, deep retinal layer; SRL, superficial retinal layer.

All cases of ADRVD involved glaucomatous eyes with central VF loss (Table 2). Additionally, the areas with ADRVD topographically corresponded to mGCL thinning and loss of central VF sensitivity (Figs. 2 and 3). Thus, ADRVD may specifically occur in glaucomatous eyes with vertically asymmetric central VF loss.

The mechanisms underlying the relationship between the development of ADRVD across the temporal raphe and functional and structural changes associated with glaucoma remain unclear; the possible concurrence of these changes further confounds the mechanism. However, while the central VF defects and mGCL thinning observed in some glaucomatous eyes topographically corresponded to ADRVD, VF loss was observed in all ADRVD cases. These findings suggested that ADRVD occurs secondary to glaucomatous structural and/or functional changes, and ADRVD results from a decreased metabolic demand of retinal ganglion cells. Nevertheless, further longitudinal studies are needed to better understand the causal relationship between ADRVD and other changes associated with glaucoma.

Interestingly, OCTA imaging has some image artefacts, such as projection artefacts and vascular shadowing. Although a post-processing algorithm has been proposed to reduce the projection artefacts^28,29^, we recognise that this algorithm is not perfect yet and the way it improves OCTA images of the deeper tissues has not been clarified. In our study, we focused on and evaluated qualitatively the asymmetrical deep vessel density loss of microvasculature within the macula area of glaucoma eyes using the PA removal software. We also confirmed the symmetrical distribution using the whole-signal-mode OCTA B-scan imaging to remove the influence of ‘false positive’ of projection artefacts. Thus, it was unlikely that symmetrical distribution of microvasculature in the superficial layer would affect the ADRVD, and we believe that the impact of PA for evaluating ADRVD was minimal.

The vascular shadowing, which was affected by the assembling superficial large retinal vessels, may cause poor visualisation of the deep retinal layer. OCTA signals are produced from variation in OCT scans; therefore, the absence of OCTA signals does not correspond to the microvasculature absence. However, the large blood vessels (i.e. arcade blood and their branch vessels that can cause shadowing in the macula area) were outside the temporal raphe area, which was the primary focus site of this study. Moreover, the whole-signal-mode OCTA B-scan images (Figs. 2j and 3k) showed superficial vascular signal reduction compared with the intact area (Figs. 2k and 3j). Thus, we believe that the effect of vascular shadowing exclusively on the specific area of the deep microvascular signal reduction was extremely low.

Although multiple OCTA studies have revealed an association between macular microvasculature changes and glaucoma, these studies mainly focused on the SRL and vessel density. Manalastas *et al*.^16^ and Ghahari *et al*.^30^ reported an association between the superficial macular VD reduction and the glaucomatous structural changes (i.e. circumpapillary retinal nerve fiber layer [cpRNFL] thickness and macular ganglion cell complex [mGCC] thickness). It has also been well documented that glaucomatous changes are related to the macular inner layer (i.e. mGCC and ganglion cell-inner plexiform layer [GCIPL]) thinning. Furthermore, Shin *et al*.^31^ showed that GCIPL thickness was lower in glaucomatous than in healthy eyes, and that this parameter was better than cpRNFL thickness for detecting parafoveal VF loss. Penteado *et al*.^22^ also reported an association between the superficial macular VD and central VF loss, and that macular VD is better than mGCC at distinguishing between glaucomatous and healthy eyes. These reports suggested that superficial macular VD is correlated with central VF sensitivity.

Interestingly, our study found a discrepancy between the affected and unaffected retinal layers regarding thickness and microvascular changes. The SRL slab in OCTA images includes the RNFL, GCL, and part of the inner plexiform layer^32^. Indeed, any GCIPL thickness change likely affects the superficial microvasculature, but the microvascular reductions were observed in the DRL despite the areas of GCL thinning topographically corresponded to those of ADRVD and central VF loss.

The underlying cause of asymmetric DRL vasculature changes remain unknown. However, we can examine the reasons that microvascular reductions were more evident in the DRL in advanced glaucomatous eyes with visual field loss threatening fixation: First, the deep vasculature is more easily affected by the ocular blood flow changes than the superficial microvasculature. Previous confocal scanning laser microscopy^33^ and OCTA^32,34^ studies have shown that capillary density is higher in the superficial than in the deep capillary layer. Spaide^35^ also reported that the deep vascular plexus was consisted by small-diameter vessels and the intravascular hydrostatic pressure would be expected to be lower than in the superficial plexus. These findings suggest that SRL is more abundant than the DRL blood flow, and that the DRL microvasculature is more vulnerable to blood flow reductions. Therefore, the DRL vessel signals may appear diminished on OCTA images before the SRL vessel signals. Second, it should be noted that the vascular measurements obtained by OCTA only reflect some aspects of blood flow within the detected vessels and estimate the actual blood flow. More specifically, this modality detects vessels using phase/Doppler shift and amplitude variation that rely upon motion within a vessel (i.e. vessel perfusion). However, OCTA does not directly quantify the blood flow rate within a detected vessel. Therefore, the observed reduction in the VD may have resulted from the capillary dropout or very slow flow rates within the perfused vessels. Third, glaucoma may have induced changes in the intermediate retinal layer, which includes Müller cells, astrocytes, and/or amacrine cells. Nützi *et al*.^36^ reported that, in eyes with glaucoma, retinal astrocytes and Müller cells were activated by ischemic or other neuronal injuries to the retina. Moreover, Ha *et al*.^37^, reported photoreceptor mitochondrial changes in the inner segment, represented as an ellipsoid zone intensity reduction on spectral-domain optical coherence tomography angiography (SD-OCTA) images, during glaucoma progression. Asaoka *et al*.^38^ also reported that the thickness of retinal pigment epithelium and outer segment layer on OCT images appears to be related to central visual sensitivity in glaucoma. This evidence, combined with the glaucoma-associated microvasculature changes suggests that glaucoma induces microvasculature damage in the intermediate and – at least in part – outer retinal layers. These are the changes that we detected as ADRVD using SS-OCTA.

In line with the results of a previous report^20^, we also found that the superficial parafoveal VD was lower in the affected than in the non- affected hemisphere, without reaching statistical significance (P = 0.092), probably because of the small sample size. We believe that our results do not entirely contradict those of these past reports. Another explanation for the discrepancy might be attributed to the differences in the type of the used OCTA machine, the analysed area, and the algorithm used for creating the OCTA image between previous works and our study. Yarmohammedi A *et al*.^20,39^ and Takusagawa HL *et al*.^29^ used a spectral-domain OCTA device and Takusagawa HL *et al*.^29^ analysed the macular microvasculature using a 6 mm ×6 mm OCTA image. Moreover, our previous study showed that the difference in the OCTA device used and the vessel density quantification method (binarisation method) affected the VD quantification^40^.

Furthermore, our study found that ADRVD more frequently occurred in eyes with an inferior than in those with a superior central VF loss, despite no significant difference between the groups in age, glaucoma severity, or systemic history. Zeiter *et al*.^41–43^ also reported a higher frequency of inferior VF defect in patients with a suspected vascular insufficiency (i.e. from diabetes). They also found that VF loss in the inferior hemifield was significantly more localised in patients with normal-tension than in those with high-tension glaucoma. These findings suggest that better understanding of these deep retinal microvascular changes, detectable with OCTA and PA removal software, may greatly enhance our understanding regarding the relationship between the glaucomatous neurodegenerative processes and DRL microvasculature reduction.

Our study had several limitations. First, nine of 43 glaucomatous eyes (20.9%) were excluded from the analyses because of the inadequate imaging (poor image quality or off-centre images) that may have introduced a selection bias. Therefore, our results should be interpreted with caution. Second, the 3 ×3 mm SS-OCTA imaging area may not have been sufficient to accurately characterise the structural-functional associations in the macula. Therefore, further studies with a larger scan area are needed. Third, several studies have already shown that systemic diseases, including diabetes, contribute to a macular VD decrease and an increase in the foveal avascular zone area^44–46^. However, an ADRVD was not detected in any of the examined patients with diabetes. Therefore, it is unlikely that diabetes heavily influenced the development of the ADRVD observed. Fourth, OCTA uses amplitude decorrelation and only detects the perfused blood vessels. Therefore, it does not provide the actual blood flow measurements, and whether the observed VD reductions reflect the capillary dropout or very slow flow rates within the perfused vessels remains uncertain. Fifth, although the SS-OCTA device, PLEX^Ⓡ^ Elite 9000, and PA removal software were commercially available and already used in a previous study^47^, the details regarding the PA removal algorithm were unclear. Therefore, further studies using other OCTA devices and PA removal software are warranted to confirm the reproducibility of ADRVD. Finally, although we were unable to provide a quantitative definition of the ADRVD in this study, the Cohen’s Kappa of intra-examiner reliability was satisfactory. Thus, the definition of ADRVD was considered acceptable for analysis.

In conclusion, we used OCTA with PA removal software to observe ADRVDs across the temporal raphe area in severe glaucomatous eyes with hemisphere central visual field loss. These vasculature changes topographically corresponded with mGCL thinning and central VF loss. These findings suggest that DRL has an important role in glaucoma development and functional progression. Further analyses of ADRVD may enhance our understanding regarding the glaucoma pathogenesis and offer new insights into its management.

## Methods

### Study design

This prospective, cross-sectional study was approved by the Ethics Committee of the Saitama Medical University (Iruma, Japan). The study adhered to the tenets of the Declaration of Helsinki, and written informed consent was obtained from each participant before any study examination was performed.

### Study participants and examinations

This study included consecutive patients with open-angle glaucoma, recruited at the Saitama Medical University Hospital between October 2017 and June 2018. All participants underwent comprehensive ophthalmic examinations, including the best-corrected visual acuity (BCVA) assessment (Landolt chart), slit-lamp biomicroscopy, IOP measurement (Goldmann applanation tonometry), fundus photography (CX-1, Canon, Inc., Tokyo, Japan), axial length measurement (Optical Biometer OA-2000, Tomey Corp., Nagoya, Japan), central corneal thickness measurement (Optical Biometer OA-2000), and SAP (Humphrey 24-2 or 30-2 Swedish Interactive Thresholding Algorithm standard [Carl Zeiss Meditec, Jena, Germany]). All participants also underwent SS-OCTA (PLEX^Ⓡ^ Elite 9000, Version 1.6.0.21130, Carl Zeiss Meditec, Jena, Germany) and SD-OCT to examine the circumpapillary retinal nerve fibre layer (cpRNFL) and mGC thickness (Spectralis HRA 2, Heidelberg Engineering, Heidelberg, Germany). The participant medical history was also acquired from the medical record, including the presence/absence of hypertension and diabetes.

Glaucoma diagnoses were made by fundus photography (finding of glaucomatous optic neuropathy, such as a vertical cup-to-disc ratio of 0.7 or higher, a rim notch with a rim width ≤ 0.1, an RNFL defect diverging in an arcuate or wedge shape, and glaucomatous VF defects based on the SAP 30-2 or 24-2 test pattern, compatible with glaucomatous optical nerve head changes. These had to fulfil at least one of the Anderson-Patella’s criteria (i.e. a cluster of ≥ 3 points in the pattern deviation plot in a single hemifield (superior/inferior) with P < 0.05, one of which must have a P value < 0.01; a glaucoma hemifield test result outside of the normal limits; or an abnormal PSD with a P value < 0.05)^48^.

Patients and eyes were excluded from the study when they metany of the following criteria: history of intraocular surgery (except for cataract or glaucoma surgery), non‌-glaucomatous optic neuropathy, vascular or nonvascular retinopathy, or other ocular or systemic disease known to impair the VF. Eyes with severe glaucoma (central VF defect in the superior and inferior hemifields or MD worse than −25 dB) were also excluded.

### Visual field classification

All glaucomatous eyes with VF defects were classified as featuring or lacking central VF loss. The latter was defined as the presence of one or more significant points in the central 10° (SITA 24-2 or 30-2 SAP algorithm) with a probability of less than 5% on the pattern deviation map. The hemifield with central VF loss was defined as the affected hemisphere. Eyes without central VF loss had clusters in the 10°–24° or 30° region with no VF loss in the central 10° region.

### Optical coherence tomography angiography examination of the macular deep microvascular reduction distribution

All SS-OCTA examinations were performed within a 3 × 3 mm (300 × 300 pixels) volume scan centred on the fovea. The SS-OCTA system had a central wavelength of 1,060 nm, an A-scan rate of 100,000 scans/s, and axial and transverse tissue resolutions of 6.0 and 20 μm, respectively. Angiography images were processed using phase/Doppler shift and amplitude variation (Optical Micro Angiography)^49^. All SS-OCTA *en face* images of the superficial retinal layer (SRL, internal limiting membrane layer to the inner plexiform layer) and the DRL (inner plexiform layer to the outer plexiform layer) were automatically obtained and analysed using built-in segmentation and the PA removal software, respectively. Where a minor segmentation error occurred, we corrected the segmentation line semi-manually. All participants underwent SD-OCT and SS-OCTA imaging on the same day. Images with severe segmentation failure, in which the segmentation line could not be corrected, with motion artefact or off-centred positioning, were excluded from the analyses. The vertical ADRVD was evaluated using SS-OCTA *en face* images of the macular DRL. An ADRVD was defined as a reduction of asymmetrical deep retinal vessel density in the DRL that was only present superior or inferior to the macular temporal raphe and did not cross the horizontal meridian.

### Parafoveal vessel density measurements

Parafoveal VD was measured as previously reported^50^. Briefly, VD was measured within an annulus with outer and inner diameters of 3 and 1 mm, respectively. Parafoveal VD was calculated for the SRL and DRL. Angiography signals were analysed with Otsu analysis^51^ for OCTA image binarization using the ImageJ software (National Institutes of Health, Bethesda, MD, USA) for obtaining the microvascular signals. The VD was, then, calculated as the percent area occupied by vessels (i.e. angiography signal area relative to the parafoveal measurement area). Parafoveal VD was also evaluated in the superior and inferior hemispheres.

### Data analyses

Data are presented as mean (standard deviation) or n (%). All visual acuity data were converted to the logarithm of the minimum angle of resolution (logMAR) prior to data analyses. Differences between groups were examined for statistical significance using the unpaired student’s t- and chi-square tests for the continuous (BCVA, IOP, axial length, spherical equivalent, MD, PSD, mGCL thickness, and cpRNFL thickness) and categorical variables (sex, hypertension, diabetes, and ADRVD), respectively. All data analyses were performed using SPSS statistical software (IBM Corp., Armonk, NY, USA), and the statistically significant level was set at *P* < 0.05. To evaluate the reliability for ADRVD determination, we measured the intra- and inter-examiner Cohen’s Kappa^52^.

## Data Availability

The datasets generated during and/or analysed during the current study are available from the corresponding author on reasonable request.
